# Radiotherapy or Surgery? Comparative, Qualitative Assessment of Online Patient Education Materials on Prostate Cancer

**DOI:** 10.3390/curroncol28050296

**Published:** 2021-09-06

**Authors:** Erwin Vu, Manolis Pratsinis, Ludwig Plasswilm, Hans-Peter Schmid, Cédric Panje, Patrick Betschart

**Affiliations:** 1 Department of Radiation Oncology, Cantonal Hospital St. Gallen, 9007 St. Gallen, Switzerland; Erwin.Vu@kssg.ch (E.V.); Ludwig.Plasswilm@kssg.ch (L.P.); cedric.panje@kssg.ch (C.P.); 2 Department of Urology, Cantonal Hospital St. Gallen, 9007 St. Gallen, Switzerland; Manolis.Pratsinis@kssg.ch (M.P.); hans-peter.schmid@kssg.ch (H.-P.S.); 3 Faculty of Medicine, University of Bern, 3008 Bern, Switzerland

**Keywords:** YouTube, patient information, online education, prostate cancer, surgery, radiotherapy

## Abstract

As multiple different treatment options are available for prostate cancer (PCa) and YouTube is commonly used as a source for medical information, we performed a systematic and comparative assessment of available videos guiding patients on their choice for the optimal treatment. An independent search for surgical therapy or radiotherapy of PCa on YouTube was performed and the 40 most viewed videos of both groups were analyzed. The validated DISCERN questionnaire and PEMAT were utilized to evaluate their quality and misinformation. The median overall quality of the videos was found to be low for surgery videos, while radiotherapy videos results reached a moderate quality. The median PEMAT understandability score was 60% (range 0–100%) for radiotherapy and 75% (range 40–100) for surgery videos. The radiotherapy videos contained less misinformation and were judged to be of higher quality. Summarized, the majority of the provided videos offer insufficient quality of content and are potentially subject to commercial bias without reports on possible conflict of interest. Thus, most of available videos on YouTube informing PCa patients about possible treatment methods are not suited for a balanced patient education or as a basis for the patient’s decision.

## 1. Introduction

Prostate cancer (PCa) is the most common noncutaneous cancer in men with over 1 million novel cancer cases worldwide and over 300,000 deaths per year [1]. Despite the availability of many consensus guidelines [2,3], the management and the choice of the suitable treatment method often poses a challenge, as multiple equivalent treatment options may be available [4,5,6]. Facing this uncertainty, the internet and online patient education materials represent an important source of information for patients [7]. With over 2 billion users monthly, YouTube is one of the most popular websites and has been demonstrated to be a commonly used source for medical information [8,9].

This shift of health-information seeking behavior could be even reinforced by the actual coronavirus disease 2019 (COVID-19) [10], which poses substantial challenges for healthcare systems worldwide [11]. In times of limited resources and social distancing [12], new healthcare services, such as consultations via telephone or video have been introduced [13]. Due to the unprecedented disruption of health care services with postponed treatments [14] online sources such as YouTube gain in importance. Accordingly, standardized methods have been introduced to evaluate the accuracy and comprehensiveness of patient education materials [15].

The specific objective of the following study was to perform a systematic and comparative assessment of available videos guiding patients on their choice for the optimal treatment of their localized PCa. While Facebook, Youtube and Twitter, as the most popular social media websites, have been shown to be used by the general public to share health information [16], we focused our search on YouTube, as the video only platform among the popular websites, which has been described to be significant for medical information, especially during the covid—pandemic [17].

## 2. Materials and Methods

An independent search for surgical therapy or radiotherapy (RT) of PCa on YouTube was performed in March 2021, in English, by four of the authors. Two of the authors were board-certified urologists (Fellow of the European Board of Urology), one author was a board-certified radiation oncologist and one a senior resident. The video search was conducted using associated keywords (see Supplementary text box). Only videos with an English audio track were included, and videos solely targeting healthcare professionals (i.e., surgical techniques, instructional explanation of device use) were excluded. Videos containing information about both therapy options were assigned to one group based on the main subject.

For each group, the 40 most viewed videos matching the inclusion and exclusion criteria were included for further analysis. The videos concerning radiotherapy were evaluated by the two radiation oncologists, while the surgery videos were assessed by the urologists.

Basic data (video upload date, length, number of views, quality of audio and video, disclosure) was assessed for every video. Further parameters documented were: type of video provider (consumer/patient; healthcare [doctor, clinic, hospital, university]; industry; news media; society/organization [foundation, governmental, academic journal]; unclear); primary and secondary topics of the video (overall procedure; surgery indications; benefits; risks/side effects; technical aspects); degree of misinformation compared to currently available evidence (no; very little; moderate; high; extreme); and depiction of real intervention (yes/no).

Every video was assessed using the validated DISCERN questionnaire [18] and examined on quality and misinformation compared to the current guidelines of the European Association of Urology (EAU) [2,3]. The DISCERN instrument was developed by an expert panel and validated by multiple major national self-help organizations. The survey consisted of 16 questions, each of which represents a separate quality criterion and is rated from one to five points (1–2 points: low; 3 points: moderate; 4–5 points: high quality). Thus, a total score of 80 points is possible, with higher scores indicating higher quality. As a second systematic evaluation, the Patient Education Materials Assessment Tool for Audiovisual Materials (PEMAT) was used [15]. The PEMAT tool was developed as a reliable and valid instrument to assess the understandability and actionability of print and audiovisual patient information. Thereby, PEMAT has been proven to demonstrate strong internal consistency, reliability, and evidence of construct validity. For our study, we utilized the version for audiovisual materials, which consist of 13 items measuring understandability and four items measuring actionability. Descriptive analysis and Mann–Whitney U tests were performed with IBM SPSS software version 27 (IBM, Armonk, NY, USA).

## 3. Results

The 80 videos included to analysis were uploaded between February 2008 (surgery)/November 2008 (radiotherapy) and September 2019 (both). The duration of the videos ranged from 35 s to 31 min. The most popular video was found in the surgery group with 4,986,282 views (1 March 2021). Table 1 shows the basic data of all videos included grouped by radiotherapy and surgery.

**Figure 1:**
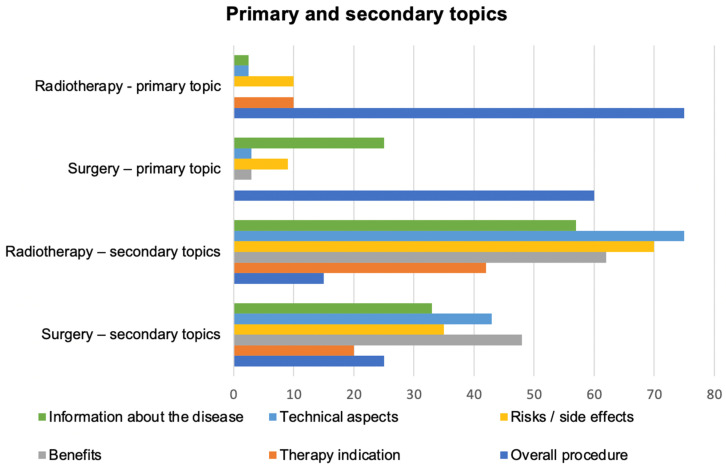
Primary and secondary topics of the videos about radiotherapy and surgery.

Most of the videos concerning radiotherapy (60%) and surgery (48%) were provided by healthcare professionals (doctor, clinic, hospital or university). Videos provided by patients were nearly three times more likely to be found in the surgery group (22%) than the radiotherapy group (7.5%). Societies (foundations, governmental institutions, academic journals) provided 20% of the radiotherapy videos and 10% of the surgery videos. Other providers (industry, news media) ranged between 5% and 15 % for both groups. The most common primary topic was the overall procedure (radiotherapy: 75%, surgery: 60%), the secondary topics of the videos varied widely between the two groups (Figure 1).

**Figure 2:**
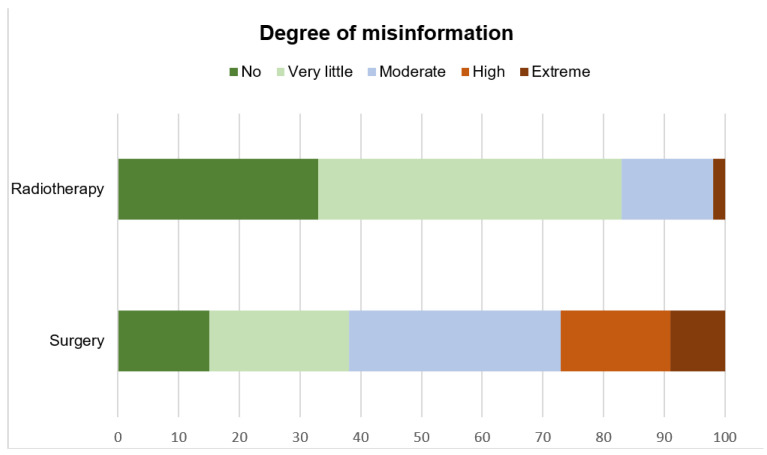
Degree of misinformation (percent, x-axis) compared to currently available evidence (no: green; very little: light green; moderate: light blue; high: light red; extreme: dark red).

Although 15% (surgery) to 23% (radiotherapy) of the videos contained commercial bias, only 10% (surgery) and 5% (radiotherapy) of the providers included a disclosure of their conflicts of interest. For both treatment modalities assessed, 19 videos (13 about radiotherapy [33%] and 6 about surgery [15%]) were rated to have no misinformation. In general, videos about radiotherapy were rated to have much less misinformation than surgery videos as demonstrated in Figure 2.

The median overall quality of the videos, according to DISCERN, was found to be low for surgery videos (2 out of 5 points for question 16; Table 2), while radiotherapy results reached a moderate quality (3 out of 5 points). Likewise, 75% of all DISCERN items reached a moderate quality for radiotherapy videos while only 31% of all DISCERN items for surgery videos were classified as moderate quality (Table 2). Adding up the results of all DISCERN questions, radiotherapy videos also resulted in a higher score (median 43 points, range 20.5–65) than the surgery videos (median 36.5 points, range 18–71). The Mann-Whitney test indicated that the median DISCERN scores were higher for the radiotherapy videos than for the surgery videos (U = 65, *p* = 0.018).

## 4. Discussion

In this study, we evaluated the quality of information of videos concerning the local treatment of PCa provided on YouTube. The RT videos were rated better than the surgery videos regarding the DISCERN questions (Table 2). In particular, RT videos were deemed to inform the viewers better about the potential benefits and risks of each treatment. Meanwhile, the surgery videos were rated higher in pointing out areas of uncertainty. In regard to understandability, the PEMAT actionability displayed similar results for both groups, with surgery videos fairing slightly better than radiotherapy videos. Noticeably, both groups failed to inform the audience about the possibility and consequences of the alternative of “active surveillance” strategies, as compared to the presented treatment method. As the keywords we used for the video search included “prostate cancer therapy” and “prostate cancer treatment” (see Supplementary text box), one would expect that the option “active surveillance” would be at least mentioned in the 80 most-viewed videos on Youtube. A quick search for videos with “active surveillance” in the title revealed only a few videos, with little views; the most-viewed video had 28,100 views, compared with 1.2 million and 4.9 million views for the most-viewed RT and surgery videos, respectively.

Overall, the RT videos contained less misinformation (Figure 2) and were judged to be of higher quality than the surgery videos. This may be in part due to the focus of RT videos on “risks/side effects” and “therapy indication” compared with the surgery videos. Remarkably, sexual dysfunction, as a side effect of surgery and reported to significantly influence patients quality of life [19,20], was rarely mentioned in the surgery videos. This lack of information may be as important as misinformation for the treatment decision-making. Furthermore, the fact that a larger part of the RT videos was provided by health care professionals and societies as opposed to patients could also be a further explanation. At least, the Mann-Whitney test showed a significant difference of median DISCERN scores between all assessed videos by professionals/societies and patient videos (U = 67.5, *p* < 0.05).

Clearly, some parts of the analyses represent subjective assessments, such as the rating of the extent of misinformation. By using the validated DISCERN and PEMAT questionnaires and the evaluation of two independent judges for each group, we attempted to minimize this subjectivity as much as possible. With the broad spectrum of quality criteria, the utilized scores are reported to be suitable methods for analyzing different consumer health-information materials [15,21,22]. To our knowledge, our analysis constitutes the first multidisciplinary analysis of an oncological condition amendable to treatment by two different specialties.

The currently available data for localized prostate cancer indicates that radiotherapy and surgery can be considered equivalent treatment options, with comparably high rates of long-term cancer control, from an oncological point of view, in most clinical scenarios. It is therefore of utmost importance to assist patients in making an informed choice regarding their preferred treatment [23]. While both methods are recommended in the current EAU guideline [2,3], the advantages and disadvantages of each treatment method differ and might influence a patient’s decision. With external-beam RT, surgery-associated complications such as bleeding, transfusion-related effects or anesthesia-associated risks could be avoided [24], however the treatment with RT is associated with a treatment course over several weeks and higher risks for rectal symptoms from radiation proctitis [25]. On the other hand, radical prostatectomy is a suitable therapy option recommended for patients with a life expectancy of at least 10 years [26,27]. In recent years, the increased use of minimally invasive surgery has led to reduced hospital time, less blood transfusions and fewer surgical complications as compared to open surgery [28]. Simultaneously, the introduction of modern image-guided RT techniques, such as volumetric modulated arc therapy (VMAT), has led to a reduction in acute gastrointestinal toxicity due to radiotherapy [29]. This brief description of the advances and the side effects caused by the different treatment methods illustrates the challenging situation for the PCa patient.

For decision-making, a detailed guidance and consultation from an interdisciplinary PCa team is crucial. The disruption of health care systems by the global COVID-19 pandemic [30,31], with its incumbent scarcity of resources [32], poses special difficulties to the fair and sufficient guidance of PCa patients [33]. While the European Association of Urology (EAU) advocated the delay of radical prostatectomy until after the pandemic [34], several studies described no outcome differences between the sequence of androgen-deprivation therapy and external-beam RT [35]. These findings also might justify the delay of prostate RT until COVID-19 infection rates decrease [36].

It is obvious that these developments and the limited availability of health care services with reduced consultations and postponed treatments [37,38] reinforce the importance of online patient-education materials. Also, the trend towards shared decision-making [39], in which patient education is a pivotal cornerstone [40], amplifie this development. Therefore, video platforms like YouTube, with large numbers of consumers, play a prominent role in patient education. While the EAU [41] and the American Urological Association (AUA) [42] each provide a YouTube channel with regularly updated content, the American Society for Radiation Oncology (ASTRO) rarely uploads videos, and with very limited reach [43]. The European Society for Radiation Oncology (ESTRO) does not have a YouTube channel, demonstrating the pent-up demand for the supply of online patient-education material of sufficient quality and information.

Our present study had some limitations. First, only English videos were included in our assessment. Second, video analyses always faced significant subjectivity (e.g., assessment of the extent of misinformation). Of particular importance is the fact, that the specialists rated the respective videos, thereby limiting the validity of a direct comparison between the groups (e.g., if one group is, in general, more critical than the other). A “cross-validation” between the two groups by the different specialists could have helped to decrease this potential bias; but, on the other hand, such an approach would weaken the fact that specialists assessed the videos of their subject, which offers advantages for the evaluation of the grade of information provided, especially where details are concerned, e.g., special RT methods. To address this limitation and to objectify the analyses, we used validated tools such as the DISCERN questionnaire and the PEMAT score. Nevertheless, the nature of video analysis remains, to a certain degree, subjective.

Our evaluation of videos concerning the treatment methods of PCa confirms the findings of previous studies [44,45]: the majority of the provided videos offer insufficient quality of content and are potentially subject to commercial bias, without reporting on possible conflicts of interest. Thereby, bias was defined as the intention to show inclinations for a treatment method instead of objectively informing the patients. In particular, the surgery videos often contained advertising for robotic-assisted surgery, a high-technology alternative which is reported to increase direct and indirect health-care costs [46]. This gives the impression that some treatment methods have been strongly marketed without balanced information about alternative methods. With this masked bias, some of the available videos on YouTube informing PCa patients about the appropriate surgical method do not qualify for a balanced patient education or as a basis for the patient’s decision. To satisfy this urge for information, the international and national societies (EAU, AUA, ASTRO, ESTRO, etc.) and local health care providers should increase their efforts and extend the offered online patient-education materials. To achieve significant reach, popular media platforms, such as YouTube, should be addressed with balanced and evidence-based information.

## 5. Conclusions

The majority of the provided videos about surgery and radiotherapy of localized PCa offer insufficient quality of content and are potentially subject to commercial bias, without reporting their possible conflict of interest. Thus, most of the available videos on YouTube informing PCA patients about possible treatment methods are not suited for a balanced patient education or as a basis for the patient’s decision.

## Figures and Tables

**Table 1:** Basic characteristic of included videos, grouped by type of treatment (radiotherapy or surgery). (Numbers are reported as median [range] unless indicated otherwise).

.	Range of Upload (Month/Year)	Median Length of Video (min:s)	Median No. of Views	Median Thumbs Up	Median Thumbs Down	Median No. of Comments	Median Audio Quality [1 (Bad)–5 (Excellent)]	Median Video Quality [1 (Bad)–5 (Excellent)]
Radiotherapy	11/2008–09/2019	06:30 [1:37–31:00]	20′848 [8077–1′291′081]	355 [20–640]	292.5 [3–582]	12 [3–21]	3.5 [3–4]	4 [3–5]
Surgery	02/2008–09/2019	04:04 [00:35–10:54]	33′123 [13′942–4′986′282]	119.5 [6–8400]	10.5 [0–1700]	13 [0–326]	5 [2–5]	5 [2–5]

**Table 2:** DISCERN single items (median [range]) for the different treatment methods assessed.

DISCERN Variable	1	2	3	4	5	6	7	8	9	10	11	12	13	14	15	16
Radiotherapy	4	3.5	3.25	3	2.5	2	2.25	2	2.5	2.5	3	1.5	2.5	2.75	3	3
	[2–5]	[1–5]	[1–5]	[1–5]	[1–5]	[1–5]	[1–5]	[1–5]	[1–5]	[1–4.5]	[1–5]	[1–0.5]	[1–5]	[1–5]	[1–5]	[1–5]
Surgery	3	3	2.5	2	2	3	2	2.5	2	2	2	1	2	2	2	2
	[2–5]	[2–5]	[1–5]	[1–5]	[1–5]	[1–5]	[1–5]	[1–5]	[1–5]	[1–5]	[1–5]	[1–5]	[1–5]	[1–5]	[1–5]	[1–5]

Color of the boxes indicate the quality of the videos: red: low (DISCERN score 1–2.4); orange: moderate (DISCERN score 2.5–3.4): green: high (DISCERN score 3.5–5). The median PEMAT understandability score was 60% (range 0–100%) for radiotherapy and 75 % (range 40–100) for surgery videos. Both therapy modalities reached a median PEMAT actionability of 50 % (range 0–100% (radiotherapy), 25–100% (surgery)).

## Data Availability

The data presented in this study are available on request from the corresponding author. The data are not publicly available.

## Supplementary Materials

The following are available online at https://www.mdpi.com/article/10.3390/curroncol28050296/s1, Supplementary text box: Key words used for video search on YouTube.
