# Supplementary material for: Radiotherapy or Surgery? Comparative, Qualitative Assessment of Online Patient Education Materials on Prostate Cancer
Source: Curr Oncol. 2021 Sep 6;28(5):3420–9. doi: 10.3390/curroncol28050296 (PMC8482178; doi:10.3390/curroncol28050296)
Supplement: Supplementary file 1 [file curroncol-28-00296-s001.zip › curroncol-1298060-supplementary.pdf]

Supplementary text box: Key words used for video search on YouTube

Radiotherapy	• Prostate cancer therapy<br>• Prostate cancer treatment<br>• Prostate cancer radiotherapy<br>• Prostate cancer external radiotherapy<br>• Prostate cancer brachytherapy<br>• External beam radiation therapy<br>• EBRT
Surgery	• Prostate cancer therapy<br>• Prostate cancer treatment<br>• Prostate cancer surgery<br>• Da Vinci surgery prostate cancer<br>• Robotic surgery prostate cancer<br>• Open surgery prostate cancer<br>• Minimal invasive surgery prostate cancer
